# Differential Effects of MitoVitE, α-Tocopherol and Trolox on Oxidative Stress, Mitochondrial Function and Inflammatory Signalling Pathways in Endothelial Cells Cultured under Conditions Mimicking Sepsis

**DOI:** 10.3390/antiox9030195

**Published:** 2020-02-26

**Authors:** Beverley E. Minter, Damon A. Lowes, Nigel R. Webster, Helen F. Galley

**Affiliations:** Institute of Medical Sciences, University of Aberdeen, Aberdeen AB41 8TJ, UK; beverley.minter1@abdn.ac.uk (B.E.M.); damon.lowes@rothamsted.ac.uk (D.A.L.); n.r.webster@abdn.ac.uk (N.R.W.)

**Keywords:** sepsis, MitoVitE, antioxidant, mitochondria, gene expression, cytokines, mRNA

## Abstract

Sepsis is a life-threatening response to infection associated with inflammation, oxidative stress and mitochondrial dysfunction. We investigated differential effects of three forms of vitamin E, which accumulate in different cellular compartments, on oxidative stress, mitochondrial function, mRNA and protein expression profiles associated with the human Toll-like receptor (TLR) -2 and -4 pathways. Human endothelial cells were exposed to lipopolysaccharide (LPS)/peptidoglycan G (PepG) to mimic sepsis, MitoVitE, α-tocopherol, or Trolox. Oxidative stress, mitochondrial function, mitochondrial membrane potential and metabolic activity were measured. NFκB-P65, total and phosphorylated inhibitor of NFκB alpha (NFκBIA), and STAT-3 in nuclear extracts, interleukin (IL)-6 and IL-8 production in culture supernatants and cellular mRNA expression of 32 genes involved in Toll-like receptor-2 and -4 pathways were measured. Exposure to LPS/PepG caused increased total radical production (*p* = 0.022), decreased glutathione ratio (*p* = 0.016), reduced membrane potential and metabolic activity (both *p* < 0.0001), increased nuclear NFκB-P65 expression (*p* = 0.016) and increased IL-6/8 secretion (both *p* < 0.0001). MitoVitE, α- tocopherol and Trolox were similar in reducing oxidative stress, NFκB activation and interleukin secretion. MitoVitE had widespread downregulatory effects on gene expression. Despite differences in site of actions, all forms of vitamin E were protective under conditions mimicking sepsis. These results challenge the concept that protection inside mitochondria provides better protection.

## 1. Introduction

Sepsis is a complex syndrome and is a leading cause of morbidity and mortality worldwide. It is characterized by a dysregulated immune response to infection, usually bacterial, leading to a systemic inflammatory response, oxidative stress, depletion of intracellular antioxidants, and ultimately organ failure [1,2]. Development of organ dysfunction associated with sepsis is now accepted to be due, at least in part, to mitochondrial dysfunction [3,4].

Vitamin E belongs to a group of compounds that includes both tocopherols and tocotrienols [5]. Vitamin E sequesters into the hydrophobic interior of membranes and α-tocopherol is the most biologically active form. Tocopherol is able to protect cell membranes from oxidation, reacting with lipid radicals produced during lipid peroxidation [5]. MitoVitE is essentially the chromanol moiety of vitamin E bound to a triphenyl phosphonium (TPP) cation and accumulates within mitochondria as a result of the large negative charge inside the mitochondrial inner membrane, with the vitamin moiety directed towards the matrix. MitoVitE has been shown to accumulate in all major organs of mice and rats after oral, intraperitoneal or intravenous administration and has potent antioxidant activity [6]. Trolox (6-hydroxy-2,5,7,8-tetra-methylchroman-2-carboxylic acid) is a synthetic, water soluble and cell-permeable derivative of vitamin E which accumulates in the cell cytosol. It is a potent antioxidant in several model systems [6,7,8,9]. 

Since mitochondria are both a major source of production and a target for damage of reactive oxygen species that contribute to oxidative stress in sepsis, antioxidants targeted to mitochondria have been proposed as a better approach than non-targeted forms for antioxidant protection in sepsis [10,11]. In this study, we investigated the relative effects of MitoVitE, α-tocopherol and Trolox on oxidative stress, mitochondrial function and expression of key genes and proteins involved in the toll-like receptor (TLR)-2 and -4 signalling pathways in human endothelial cells cultured in an environment mimicking acute bacterial sepsis. Despite differences in site of actions, we found that all three forms of vitamin E had protective effects in human endothelial cells under conditions mimicking sepsis.

## 2. Materials and Methods 

### 2.1. Chemicals 

Unless otherwise stated, all chemicals were obtained from Sigma-Aldrich (Poole, Dorset, UK)

### 2.2. Cell Culture

The human umbilical vein endothelial cell line (HUVEC-C, obtained from ATCC, Teddington, Middlesex, UK) was used from passages 7 to 10 as described previously in detail [12,13,14]. Microscopy images of the cells are shown in Supplementary Figure S1. For experimentation, cells were cultured in 96- or 6-well plates in the presence of 0.2 µg/mL lipopolysaccharide (LPS) plus 20 µg/mL peptidoglycan G (PepG) plus 5 µM MitoVitE, α-tocopherol acetate or Trolox or molecular grade ethanol as vehicle control. The duration of treatment was either 4 h, 24 h or 7 d based on expected expression profiles as detailed below. Cell viability was assessed using acid phosphatase activity [15]. 

### 2.3. Oxidative Stress

Total radical production was measured in intact cells as follows: following treatment for 24 h, cells were washed with PBS before being loaded with 50 µM of the oxidation sensitive dye 5-(-6)-carboxy-2’,7′-dichlorofluorescein diacetate (carboxy-DCFDA, molecular probes, Invitrogen, Paisley, UK) in Hank’s balanced salt solution (HBSS) supplemented with 1g/L glucose, and incubated for 1 h in the dark at 37 °C. Following incubation, cells were washed with phosphate buffered saline (PBS, pH 7.4) and fluorescence was determined immediately over 3 h at 37 °C at an excitation wavelength of 485 nm/emission wavelength 530 nm.

For measurement of reduced glutathione (GSH), buffer containing 0.1% (*v*/*v*) Triton X-100 and 0.1 M potassium phosphate, pH 6.5, plus 20 μM monochlorobimane was added to cells after 24 h treatment, for 37 °C in the dark for 30 min. To measure oxidised glutathione (GSSG), buffer containing 0.1% (*v*/*v*) Triton X-100 and 0.1 M potassium phosphate, pH 6.5 and 2.2 mM diethylenetriamine-penta-acetic acid and 2 mM dithiothreitol were added to additional cells for 30 min at 37 °C followed by the addition of an equal volume of 40 μM monochlorobimane for 30 min at 37 °C. Fluorescence was determined at ambient room temperature (excitation 355 nm, emission 520 nm) and data were normalised to total cellular protein level, determined using the Bradford method [16]. 

### 2.4. Mitochondrial Function

Mitochondrial membrane potential was determined in intact cells using the fluorescent probe JC-1 (5,5,6,6-tetrachloro-1,1,3,3-tetraethylbenzimidazolcarbocyanine iodide (Invitrogen, Paisley, UK) [12,13,14]. Following treatments for 7 d, with a medium change after 3–4 days, cells were washed with PBS, incubated for 30 min with 10 µg/mL JC-1 in PBS at 37 °C in the dark, then washed with PBS and the red/green fluorescence ratio measured. A decrease in the ratio of red/green fluorescence indicates loss of mitochondrial membrane potential [12,13,14]. As a positive control, some cells were treated with 1 µM rotenone without LPS/PepG for 5 h prior to the addition of JC-1. Metabolic activity was analyzed by measuring the rate of reduction of Alamar Blue™ (Invitrogen, Paisley, UK) in intact cells after 7 d treatment as described above. Alamar Blue™ is a novel redox indicator that exhibits both fluorescent and colourimetric changes in response to changes in metabolic activity via oxidative metabolism [17]. Briefly, Alamar Blue^TM^ was added to each well and fluorescence was measured every 15 min for 2 h at 37 °C at excitation 530 nm/emission 620 nm. Metabolic activity was determined as the rate of change in fluorescence over time [13,14]. 

### 2.5. Differential Gene Expression

The genes were selected based on their roles in the TLR2 and TLR4 pathways which ultimately result in activation of nuclear factor kappa B (NFκB). This transcription factor is known to be redox sensitive and has a crucial role in propagation of the inflammatory response to sepsis. The TLR2 and TLR4 signalling pathways are illustrated in Supplementary Figure S2 and the genes investigated are listed in Supplementary Table S1. Expression of 32 key genes was measured using pre-coated custom RT-PCR plates (Applied Biosystems, Warrington, UK). 

Total RNA was isolated from treated cells after 4 h treatments using Trizol and further purified using the Qiagen RNeasy Mini Kit with on-column genomic DNA digestion. Total RNA (100ng) was used for cDNA synthesis using the Applied Biosystems high capacity RNA-to-cDNA TaqMan^®^ Kit. For qPCR, cDNA was mixed with TaqMan^®^ gene expression master mix (Applied Biosystems, Warrington, UK) and added to the pre-coated well plate. Targets were amplified and detected using the 7500 HT Fast Real-Time PCR System. Hypoxanthine-guanine phosphoribosyltransferase-1 (HPRT-1) was used as housekeeping gene. The PCR system run cycle: activation (95 °C, 10 min), melt (95 °C, 15 s), annealing and extension (60 °C, 1 min) for 40 cycles. Gene expression (as fold change) was determined using the Delta-Delta C_t_ (2^−ΔΔCT^) method using the SA Biosciences (Qiagen, Manchester, UK) web-based PCR array system from raw threshold cycle data (C_t_). The ΔΔC_t_ algorithm is an approximation method to determine relative gene expression with quantitative real-time PCR (qPCR) experiments. Six independent experiments were performed and a minimum fold change of at least 2 compared to LPS/PepG treatment alone was pre-defined. *p* values were calculated using Student’s T-tests of the replicate 2^−ΔΔCT^ values for each gene and a *p* value of ≤0.05 was taken as significant. 

### 2.6. Protein Expression

To determine NFκB activation, nuclear extracts from treated cells were prepared following 4 h treatments using the Novagens Nucbuster^TM^ protein extraction kit (Merck Chemicals Ltd., Nottingham, UK). NFκB activation was measured as the amount of the p65 subunit present in the nucleus using the Novagen NoShift^TM^ transcription factor assay kit (Merck, Nottingham, UK) [12,13]. To determine phosphorylated inhibitor of NFκB alpha (NFκBIA, also known as IκBα), and signal transducer and activator of transcription-3 (STAT-3) activation, after 4 h exposure to LPS/PepG, cells were lysed in TRIS Base buffer containing protease/ phosphatases inhibitors and adjusted to a protein concentration of 0.25 mg/mL. Commercially available enzyme immunoassay kits were used to quantify the total and phosphorylated proteins (InstantOne^TM^ eBioscience Ltd., Hatfield, UK) according to the manufacturer’s protocols. Commercially available enzyme immunoassay kits were used to quantify interleukin (IL)-6 and IL-8 secretion (R&D Systems, Oxford, UK) in culture supernatants of cells treated with LPS/PepG with and without the three forms of vitamin E for 24 h, as described in the manufacturer’s protocol. 

### 2.7. Statistical Analysis

For oxidative stress and mitochondrial function assays, six independent experiments were performed (*n* = 6). For protein expression, 3–6 independent experiments were performed. No assumptions were made about data distribution. Data were analysed using non-parametric Kruskal Wallis testing with Mann Whitney post hoc testing where appropriate and are presented as median, interquartile and full range, or individual raw data points when *n* ≤ 6. A *p* value of ≤ 0.05 was taken to be significant. 

## 3. Results

### 3.1. Cell Viability

Acid phosphatase activity was similar regardless of cell treatment at both 24 h and 7 d, showing no detrimental effect on cell viability (Supplementary Figure S3).

#### 3.1.1. Oxidative Stress

**Figure 1:**
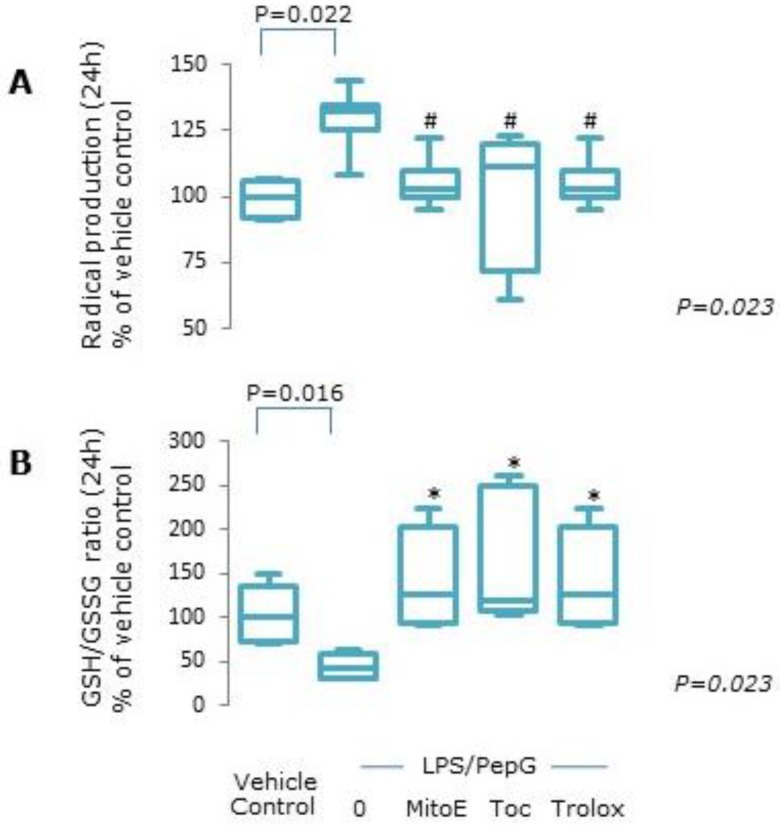
Oxidative stress. Endothelial cells were treated with vehicle control, lipopolysaccharide plus peptidoglycan (LPS/PepG) alone, or LPS/PepG plus 5 μM MitoVitE (MitoE), tocopherol (Toc) or Trolox for 24 h. (**A**) Total radical production, (**B**) reduced/oxidised glutathione. Box and whisker plots show median, interquartile and full range (*n* = 6). *p* value in italics refers to Kruskal–Wallis across LPS/PepG treated groups. # = significantly lower and * = significantly higher, than LPS/PepG alone (*p* < 0.05).

Exposure of endothelial cells to LPS/PepG resulted in a significant increase in total radical production compared to vehicle control (*p* = 0.022, Figure 1A). Co-treatment of cells with any of the forms of vitamin E plus LPS/PepG abrogated the increase in radical production (Figure 1A). The ratio of GSH:GSSG was significantly lower in LPS/PepG treated cells compared to vehicle control treated cells (*p* = 0.016, Figure 1B). Co-exposure to LPS/PepG in the presence of all of the forms of vitamin E prevented the LPS-PepG mediated decrease in the glutathione ratio (Figure 1B). 

#### 3.1.2. Mitochondrial Function 

**Figure 2:**
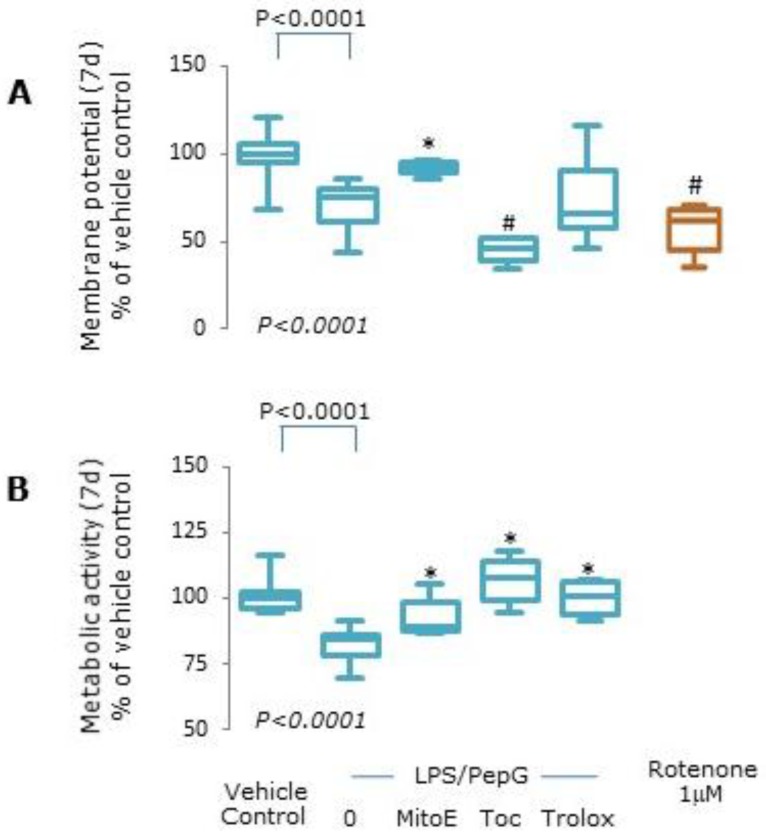
Oxidative stress. Endothelial cells were treated with vehicle control, lipopolysaccharide plus peptidoglycan (LPS/PepG) alone, or LPS/PepG plus 5 μM MitoVitE (MitoE), tocopherol (Toc) or Trolox for 7 d, or 1μM rotenone. (**A**) Mitochondrial membrane potential, (**B**) metabolic activity. Box and whisker plots show median, interquartile and full range (*n* = 6). *p*-value in italics refers to Kruskal–Wallis across LPS/PepG treated groups. # = significantly lower and * = significantly higher, than LPS/PepG alone (*p* < 0.05).

Cells exposed to LPS/PepG for 7 d had significantly lower mitochondrial membrane potential compared to vehicle control treated cells (*p* < 0.0001, Figure 2A). Membrane potential was significantly higher in cells exposed to LPS/PepG plus MitoVitE compared to LPS/PepG alone (*p* = 0.003) but not in those cells treated with Trolox or α-tocopherol; indeed, tocopherol worsened the loss of membrane potential (Figure 2A). Pre-treatment with rotenone resulted in around 50% loss of membrane potential in vehicle control treated cells (Figure 2A). 

Metabolic activity was also significantly lower in cells exposed to LPS/PepG compared to vehicle control cells (*p* < 0.0001) and co-treatment with MitoVitE (*p* = 0.03), Trolox (*p* = 0.002) or α-tocopherol (*p* = 0.002) ameliorated the loss of metabolic activity (Figure 2B). 

#### 3.1.3. Gene Expression 

**Figure 3:**
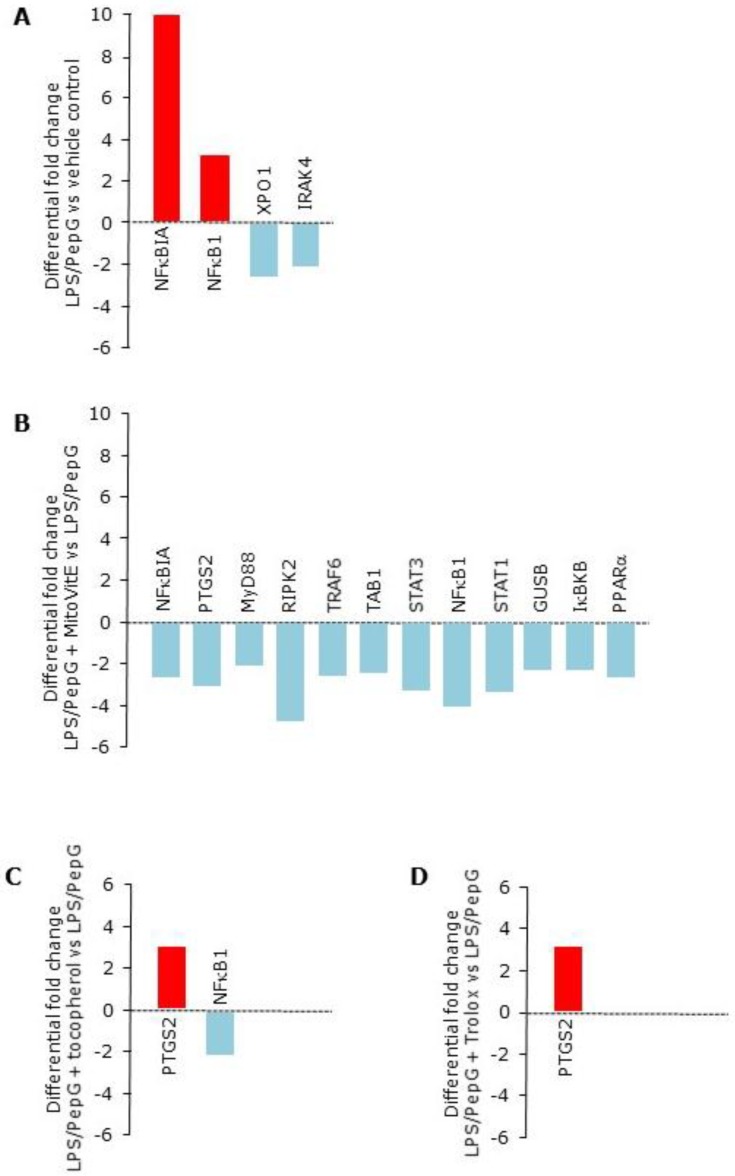
Gene expression. Differential gene expression as mean fold change in endothelial cells showing the effect of (**A**) Lipopolysaccharide plus peptidoglycan (LPS/PepG) compared to vehicle control (**B**) LPS/PepG plus 5 μM MitoVitE compared to LPS/PepG alone (**C**) LPS/PepG plus 5 μM tocopherol compared to LPS/PepG alone or (**D**) LPS/PepG plus 5 μM Trolox compared to LPS/PepG alone. Bars show mean fold changes where *p* < 0.05. Red = upregulation, blue = downregulation, *n* = 6. 95% confidence intervals and *p* values are given in Table 1.

Identities of the 32 genes analysed and their associated proteins and functions are summarized in Supplementary Table S1 and Supplementary Figure S2. Differential gene expression following LPS/PepG exposure for 4 h compared to vehicle control showed that expression of 7 genes were upregulated by at least 2–fold but only two of these were statistically significant: NFκBIA (*p* = 0.02) and NFκB1 (*p* = 0.04, Table 1, Figure 3A). Two genes were downregulated by >2 fold (XPO1 and IRAK4, both *p* = 0.02, Table 1, Figure 3A). In cells exposed to LPS/PepG plus MitoVitE, no genes were upregulated, but 12 genes were downregulated compared to LPS/PepG alone (*p* < 0.05, Table 1, Figure 3B). In contrast, only one gene, NFκB1, was downregulated in cells exposed to LPS/PepG plus α-tocopherol (*p* = 0.002, Table 1, Figure 3C) but none were downregulated by Trolox. PTGS2 was upregulated by α-tocopherol and Trolox (*p* = 0.02 and *p* = 0.006 respectively), a gene which was downregulated by MitoVitE (*p* = 0.02, Table 1, Figure 3D).

#### 3.1.4. Protein Expression

**Figure 4:**
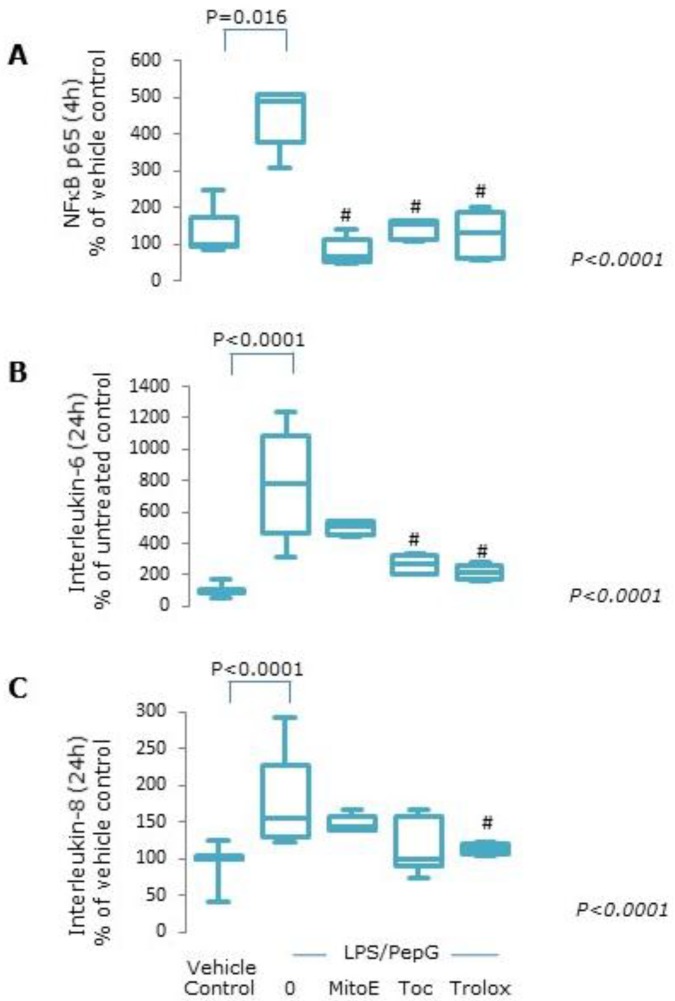
NFκB activation and cytokine expression. Endothelial cells treated with vehicle control, lipopolysaccharide plus peptidoglycan (LPS/PepG) alone, or LPS/PepG plus 5 μM MitoVitE (MitoE), tocopherol (Toc) or Trolox for 4 or 24 h. (**A**) Nuclear NFĸB p65, (**B**) interleukin-6 (IL-6) and (**C**) interleukin-8. Box and whisker plots show median, interquartile and full range (*n* = 6). *p* value in italics refers to Kruskal–Wallis across LPS/PepG treated groups, # = significantly lower than LPS/PepG alone (*p* < 0.05).

Nuclear NFκB-p65 protein was maximally expressed following 4 h LPS/PepG exposure (Supplementary Figure S4) and was higher than vehicle control treated cells (*p* = 0.016, Figure 4A). Co-treatment of cells with either of the three forms of vitamin E plus LPS/PepG resulted in decreased nuclear NFκB-p65 protein expression similar to that seen in vehicle control treated cells (Figure 4A). Significant increases in both IL-6 and IL-8 protein levels were seen in culture supernatants from cells exposed to LPS/PepG for 24 h compared to vehicle control treated cells (both *p* < 0.0001, Figure 4B,C). All forms of vitamin E appeared to suppress IL-6 and IL-8 secretion when compared to LPS/PepG exposed cells. However, IL-6 levels were statistically significantly lower only in cells exposed to LPS/PepG plus α-tocopherol or Trolox compared to LPS/PepG alone (Figure 4B) and IL-8 levels were significantly lower only in Trolox treated cells (Figure 4C). 

**Figure 5:**
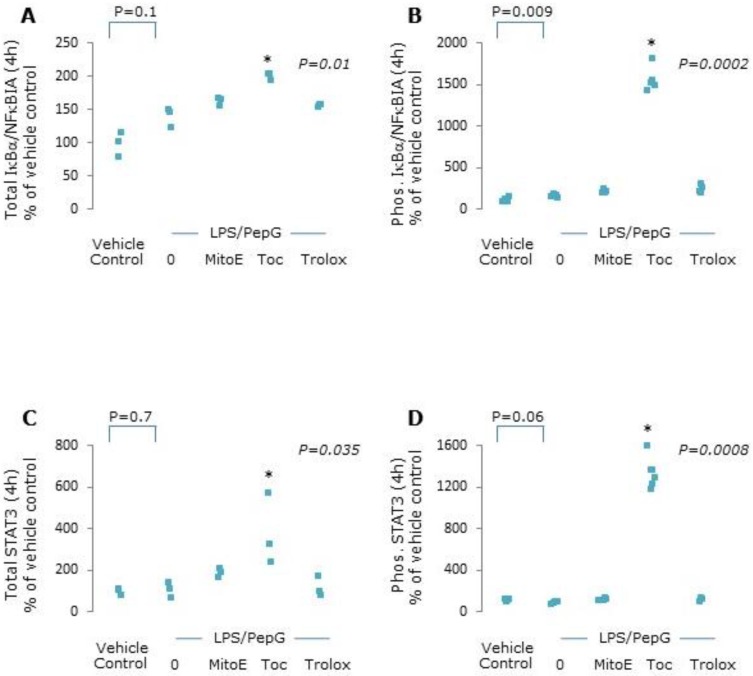
Total and phosphorylated IκBα and STAT3 expression. Endothelial cells were treated with vehicle control, lipopolysaccharide plus peptidoglycan (LPS/PepG) alone, or LPS/PepG plus 5 μM MitoVitE (MitoE), tocopherol (Toc) or Trolox for 4 h. (**A**) Total IκBα, (**B**) phosphorylated IκBα, (**C**) total STAT3 and (**D**) phosphorylated STAT3. Individual data points shown (*n* = 3–6). *p* value in italics refers to Kruskal–Wallis across LPS/PepG treated groups, * = significantly higher than LPS/PepG alone (*p* < 0.05).

Total and phosphorylated NFκBIA and STAT3 proteins were markedly increased in cells co-treated with LPS/PepG and α-tocopherol, whilst MitoVitE and Trolox had no effect (Figure 5).

## 4. Discussion

We found that exposure of human endothelial cells to LPS/PepG resulted in mitochondrial dysfunction and oxidative stress, associated with changes in expression of genes and selected proteins involved in TLR2 and TLR4 signalling and the inflammatory response to conditions of sepsis. Surprisingly, we found that although the effects of the three forms of vitamin E act in different cellular compartments, we found largely similar effects on mitochondrial function and oxidative stress. However, only the mitochondria targeted form of vitamin E, MitoVitE, had widespread downregulatory effects on genes involved in TLR pathways, resulting in a clear anti-inflammatory profile.

Oxidative stress and mitochondrial damage/dysfunction are consistent findings in sepsis and drive the dysregulated and prolonged inflammation seen in these patients [3,4]. TLRs are pattern recognition receptors which detect pathogen associated molecular patterns (PAMPs) that include LPS and PepG, and initiate signal transduction, culminating in the activation of NFκB and transcription of cytokines [18,19]. Exposure of endothelial cells to LPS/PepG resulted in maximal nuclear translocation of NFκB at 4 h. Since antioxidants acting in mitochondria have been advocated as a better protective strategy in sepsis than those that do not [10,11], and as NFκB has a fundamental and wide ranging role in propagation of immune responses in sepsis [20], we determined the effects of compartmentalized antioxidants on expression of genes involved in relevant pathways (see Supplementary Figure S2) at maximal NFκB protein expression and subsequent downstream effects on inflammatory mediator expression and mitochondrial function. Many studies report on the expression of genes known to be regulated by NFκB following PAMP-induced activation [18,19,20,21]. NFκB comprises DNA-binding subunits, principally NFκB1 and NFκB2, in heterodimers with transcriptional activators including P65, also known as RelA [18]. Inactivated NFκB complexes are retained in the cellular cytoplasm by inhibitors including NFκBIA. The process of NFκB activation involves translocation of NFκB heterodimers into the nucleus and activation of the transcriptional subunit, which is initiated by phosphorylation, resulting upregulation of a large number of genes required for host immune and inflammatory mediators, including chemokines, cytokines, enzymes and adhesion molecules. It is known that complete activation of NFκB requires a redox sensitive step and so antioxidants may have a role in its regulation [18,19]. However, effects on the signalling cascades leading to NFκB activation are less clear.

We and others have consistently reported low serum tocopherol levels and elevated oxidative stress biomarkers in patients with sepsis [22,23,24,25]. Although all tocopherols and tocotrienols have pronounced antioxidant activity in model systems in vitro, there are variable reports of the effect of administration of vitamin E on biomarkers of oxidative stress and there is little evidence of measurable metabolites of vitamin E after antioxidant reactions in vivo [26,27]. It has been shown that α-tocopherol, the most biologically relevant tocopherol, inhibits the signalling cascades initiated by LPS in neutrophils and microglial cells in vitro [28] and inhibits LPS- induced inflammatory responses in murine macrophages in vitro [29]. MitoVitE contains a 2-carbon chain which links the functional chromanol moiety of vitamin E to a TPP cation and is able to cross the outer mitochondrial membrane due to the positive charge of the TPP conjugate [6,30]. In Trolox, the chromanol is connected to a short-chain carboxylic acid making Trolox hydrophilic and cell-permeable such that it accumulates in the cell cytosol [6,7,8,9]. MitoVitE is more effective than Trolox both in vitro and in vivo [6,31]. Antioxidants which specifically accumulate within the mitochondrial matrix are suggested to offer better protection during sepsis-induced oxidative stress than non-targeted antioxidants, since mitochondria are the main site of radical production during inflammation [10,11]. We have shown that antioxidants which accumulate inside mitochondria are protective against mitochondrial dysfunction in human endothelial cells exposed to conditions which mimic those seen in sepsis [13,32] and have beneficial effects in an animal model of acute sepsis [32,33]. The present study was undertaken to determine the differential effects of compartmentalization of antioxidants using α-tocopherol, MitoVitE and Trolox, on mitochondrial function, oxidative stress and gene expression of proteins which are involved in the TLR2/4 signalling pathways, in an in vitro model relevant to sepsis. We have reported previously on this model [12,13,14,22,32].

Exposure of endothelial cells to LPS/PepG in vitro results in consumption of glutathione and initiation of inflammatory responses associated with activation of NFκB and mitochondrial oxidative stress [12,13,14,22,32]. We also found that following exposure of endothelial cells to LPS/PepG there was increased production of intracellular radicals, which was abrogated when the cells were cultured with all three forms of vitamin E tested. Alpha-tocopherol has been reported to reduce intracellular radical production in several other oxidative stress models [28,29] and Trolox is an effective intracellular radical scavenger in human cells [7,8]. We also showed that the three vitamin E derivatives ameliorated the reduction in the glutathione ratio caused by exposure to LPS/PepG. Only MitoVitE was able to prevent the loss of mitochondrial membrane potential, although all three compounds were able to maintain mitochondrial metabolic activity after LPS/PepG exposure of cells. This suggests that loss of membrane potential is an adaptive response to prevent an increase in radical formation by increasing uncoupling protein-2 to uncouple the electron transport chain [34]. Our results suggest that the loss of metabolic activity is either caused by damage to components of the tricarboxylic acid cycle and/or mitochondrial oxidative phosphorylation complexes with no substantial reduction of ATP synthesis since the resultant increase in AMP production would either drive an increase in metabolic activity, or again is an adaptive response in this model to limit damage. We have reported previously that other mitochondria targeted antioxidants are protective in this model [32,34]. We have also shown mitochondrial protective effects of MitoVitE in dorsal root ganglion cells exposed to the chemotherapy drug, paclitaxel, where MitoVitE but not Trolox was able to protect against mitochondrial dysfunction and loss of GSH [31]. Only MitoVitE is able to scavenge radicals at source and ameliorate mitochondrial uncoupling, as shown by maintenance of membrane potential.

NFκB-P65 subunit expression was maximal at 4 h following LPS/PepG exposure. We found significant upregulation, consistent with other studies, of NFκB-P50 mRNA in LPS/PepG exposed cells, whose protein is known to modulate NFκB activity, and NFκBIA, which encodes for an inhibitor subunit of NFκB; this may be a negative regulatory process to dampen the inflammatory response. Following phosphorylation, NFκBIA is ubiquinitated and degraded, which results in an increase in NFκB activity. However, the increase in NFκBIA phosphorylation was not reflected by p65, IL-6 or, IL-8 levels. Two genes were differentially downregulated by LPS/PepG exposure: XPO1 and IL-1 receptor-associated kinase 4 (IRAK4). XPO1 encodes for exportin-1 which is involved in trafficking of proteins including NFκBIA [35,36] whereas IRAK4 is a key mediator in TLR2 and -4 signalling [37]. Overexpression of XPO1 results in increased translocation of active NFκB [35].

The increase in LPS/PepG-induced NFκB-p65 translocation was abrogated by all forms of vitamin E tested, concomitant with lower IL-6 and IL-8 secretion. NFκB1 gene expression was also downregulated by MitoVitE and tocopherol, but not Trolox. Tocopherols and tocotrienols and a synthetic chroman carboxamide with a chemical structure similar to Trolox were previously shown to reduce LPS-induced NFκB activation and cytokine secretion [28,29,38,39]. In vitro, MitoVitE reduced NFκB nuclear translocation [40] and in vivo dampened cytokine responses and improved sepsis-induced organ dysfunction [33,41]. We also found higher total and phosphorylated STAT3 protein expression in cells treated with LPS/PepG plus tocopherol but not MitoVitE or Trolox. STAT3 is a transcription factor activated in response to cytokines by receptor associated Janus kinases and MAPK [42] which can localize within the mitochondria and is a regulator of the electron transport chain [43]. Suppression of STAT3 by tocopherols and tocotrienols has been reported (reviewed by Jiang in [5]) but the mechanism of changes in NFĸBIA and STAT3 protein seen here with tocopherol are unclear. However, gene expression of STAT3 following tocopherol treatment remained similar to LPS/PepG and suggests modulation by tocopherol at the post-translational level.

Analysis of gene expression showed that endothelial cells exposed to either α-tocopherol or Trolox plus LPS/PepG resulted in upregulation of inducible prostaglandin endoperoxide synthase 2 (PTGS2, also known as COX-2). This protein is involved in prostanoid biosynthesis and is a mediator of the inflammatory response. In contrast, this gene was downregulated by MitoVitE compared along with several other crucial components of the TLR signalling cascade which. These include IκBKB, NFκB1, MyD88 and TRAF6 (see Supplementary Table S1 for functional roles). These genes encode for proteins that interact to initiate downstream signalling to NFκB (see Supplementary Figure S2). Downregulation of these genes indicate a clear anti-inflammatory profile for MitoVitE. In addition, STAT1 and STAT3 mRNA were also downregulated by MitoVitE. STAT1 has been shown to be involved in interferon signalling and cell survival. Many of these genes downregulated by MitoVitE have been shown to be upregulated in mononuclear cells from patients with sepsis [20]. It is intriguing that the functional effects of all three antioxidants on LPS/PepG-induced transcriptional activation, cytokine expression and mitochondrial function were broadly similar, yet the effects at the mRNA level were very different. It has been suggested that some of the anti-inflammatory effects of tocopherol are independent of its antioxidant activity [44]. Plasma membrane lipid rafts containing tocopherol can interact with TLRs and may be important in the response to LPS exposure and sepsis [45,46].

## 5. Conclusions

This was an in vitro study using human endothelial cells under conditions designed to mimic the changes seen in sepsis. As such is one of several tools for translational experimental approaches to investigate potential therapeutic strategies in patients with sepsis. Since the primary site of free radical production in sepsis is inside mitochondria, we expected that antioxidants that acting elsewhere in the cell may be less effective than those acting inside mitochondria. We did not expect to find that tocopherol and Trolox would be as functionally effective as MitoVitE at blunting the inflammatory consequences of key mediators involved in the response to LPS/PepG exposure, despite more marked anti-inflammatory effects of MitoVitE at the gene expression level. These results challenge current thinking and warrant further investigation.

## Figures and Tables

**Table 1:** Differential gene expression.

Gene Name.	LPS/PepG ^a^	LPS/PepG	LPS/PepG	LPS/PepG
		+ MitoVitE ^b^	+ α-Tocopherol ^b^	+Trolox ^b^
**GUSB**		−2.5		
		(0.27, 0.52)		
		*p* = 0.05		
**IĸBKB**		−2.5		
		0.25, 0.56		
		*p* = 0.017		
**IRAK4**	−2.2			
	(1.05, 3.42)			
	*p* = 0.02			
**MYD88**		−2.4		
		(0.23, 0.61)		
		*p* = 0.05		
**NFκB1**	+4.4	−4.5	**−3.9**	
	(0.06, 0.40)	(0.17, 0.28)	(0.18, 0.51)	
	*p* = 0.01	*p* = 0.0003	*p* = 0.002	
**NFĸBIA**	+11.7	-2.7		
	(0.00001, 0.18) 0.18)	(0.18, 0.56)		
	*p* = 0.02	*p* = 0.04		
**PPAR-α**		−3.0		
		(0.17, 0.50)		
		*p* = 0.02		
**PTGS2**		-3.5	**+3.0**	**+2.9**
		(0.12, 0.45)	(1.29, 4.62)	(1.30, 4.50)
		*p* = 0.02	*p* = 0.005	*p* = 0.006
**RIPK2**		−5.4		
		(0.12, 0.25)		
		*p* = 0.001		
**STAT1**		−3.7		
		(0.20, 0.34)		
		*p* = 0.0002		
**STAT3**		−3.1		
		(0.25, 0.40)		
		*p* = 0.0002		
**TAB1**		−2.8		
		(0.23, 0.49)		
		*p* = 0.009		
**TRAF6**		−2.9		
		(0.24, 0.44)		
		*p* = 0.0003		
**XPO1**	−2.7			
	(0.98, 4.33)			
	*p* = 0.02			

^a^ Mean fold change (red), 95% confidence interval (CI) and *p*-value compared to vehicle control or ^b^ to LPS/PepG alone. CI = indicateS 95% certainty that the mean value is the true mean of the population. For full gene names and functions see Supplementary Table S1.

## Supplementary Materials

The following are available online at https://www.mdpi.com/2076-3921/9/3/195/s1, Figure S1: Microscopy images. Figure S2: Simplified Toll like receptor signaling pathway, Figure S3: Acid phosphatase activity, Figure S4: Western blot of nuclear NFĸB P65 protein expression, Table S1: Gene ID and function.
