# Supplementary material for: Differential Effects of MitoVitE, α-Tocopherol and Trolox on Oxidative Stress, Mitochondrial Function and Inflammatory Signalling Pathways in Endothelial Cells Cultured under Conditions Mimicking Sepsis
Source: Antioxidants (Basel). 2020 Feb 26;9(3):195. doi: 10.3390/antiox9030195 (PMC7139367; doi:10.3390/antiox9030195)
Supplement: Supplementary file 1 [file antioxidants-09-00195-s001.zip › Supplementary Table 1.pdf]

Supplementary Table 1. Gene ID and function

Gene ID	Full gene name	Summary of functions
**CD14**	Cluster of differentiation 14	Essential for TLR4 immune response. Binds to bacterial LPS and delivers it to MD-2/TLR 4 complex. Role in activation of MyD88, TIRAP and TRAF 6, leading to NFκB activation and cytokine secretion.
**CEBPB/ NF-IL-6 beta**	Nuclear factor IL-6	Specifically binds to an IL-1 response element in the IL-6 gene. Binds to regulatory regions of several acute-phase and cytokines genes. Interacts with PTGES2.
**GUSB**	Glucuronidase beta	Hydrolase that degrades glycosaminoglycans.
**IFNB1**	Interferon beta 1	Early innate immune response cytokine
**IRAK1**	Interleukin-1 receptor-associated kinase 1	Has a role in IL-1 receptor activation. Binds to MyD88 which phosphorylates IRAK1 by IRAK4 and leads to NFκB activation.
**IRAK4**	Interleukin-1-receptor-associated kinase 4	Forms a complex with MyD88 and IRAK2. Phosphorylated IRAK4 associates with TRAF 6 and TIRAP and PELI1 this intermediate complex is required for NFκB activation.
**IκBKB**	Inhibitor of nuclear factor kappa B kinase subunit beta	Critical role in the NFκB pathway as an enzyme complex of the canonical IKK complex. Phosphorylates inhibitors of NFκB on 2 critical serine residues and degrades them by the proteasome to allow NFκB translocation into the nucleus.
**MAP2K1**	Mitogen-activated protein kinase kinase 1	Important component of the MAP kinase signal transduction pathway, binds cytokine ligands.
**MAP3K1**	Mitogen-activated protein kinase kinase kinase 1	A stress activated serine/threonine kinase ,plays a role in signal transduction cascades of the JNK kinase and the NFκB pathway.
**MAPK8**	mitogen-activated protein kinase 8	Mediates early gene expression in response to LPS and pro-inflammatory cytokines and regulates JNK signalling

**MAPK8IP3**	Mitogen-activated protein kinase 8 interacting protein 3	Role in the function of JNK signalling as a scaffold protein for MAPK cascade.
**MYD88**	Myeloid differentiation primary response gene 88 also known as TIRAP	Adaptor protein involved in TLR2 and -4 and IL-1 signalling. Acts via IRAK1, IRAK2, IRF7 and TRAF 6, leading to NFκB activation, cytokine secretion and IL-8 transcription.
**NFκB1**	Nuclear factor of kappa light polypeptide gene enhancer in B-cells 1	Has dual function of cytoplasmic retention of NFκB proteins by p105 and generates p50 by co-translational processing.
**NFκBIA**	Nuclear factor of kappa light polypeptide gene enhancer in B-cells inhibitor, alpha	Retains the NFκB complexes in the cytoplasm until required in the nucleus. Also known as IκBα.
**PPAR-α**	Peroxisome proliferator-activated receptor alpha	Involved in the immune and inflammation responses and regulates fatty acid synthesis, oxidation and lipoprotein assembly.
**PTGS2**	Prostaglandin-endoperoxide synthase 2	Inducible key membrane protein in prostaglandin biosynthesis. NFκB can also induce PTGST2 expression. Also known as cyclo-oxygenase 2 (COX2).
**RIPK2**	Receptor interacting serine/ threonine kinase 2	Potent activator of NFκB especially by peptidoglycans. Activates tyrosine phosphorylation that leads to NFκB activation by NOD2.
**SOCS1**	Suppressor of cytokine signalling 1	A negative regulator of cytokine signalling through the JAK/STAT3 pathway. Major regulator of IL-6.
**SOCS3**	Suppressor of cytokine signalling 3	A negative regulator of cytokine signalling through the JAK/STAT3 pathway by binding to tyrosine kinase receptors including gp130. Induced by various cytokines, including IL-6.
**STAT1**	Signal transducer and activator of transcription 1	Protein is phosphorylated by the receptor-associated kinases, translocates to the nucleus, and acts as transcription activator. Role in the activation of JAK kinases.
**STAT3**	Signal transducer and activator of transcription 3	Part of JAK-STAT signalling cascade and are phosphorylated by receptor associated kinases in responses to IL-6. Localised in mitochondria.

**TAB1**	TGF-beta-activated kinase 1/map3k7-binding protein	Regulator of MAP3K pathway mediating intracellular signalling induced by TGF-β, IL-1. Interacts with TRAF 6 and MAP3K7 leading to NFκB activation
**TBK1**	Tank binding kinase 1	Involved in activation of TLRs by bacteria. Serine/threonine kinase with essential role in regulating inflammatory responses by mediating NFκB activation through phosphorylation of serine.
**TICAM1**	Toll like receptor adaptor molecule 1 (also known as TRIF)	Facilitates protein-protein interactions for TLR4 and signal-transduction constituents, Recruits proteins TBK1, TRAF6 and RIPK1 which activates NFκB
**TICAM2; TMED7-TICAM2**	Tmed7-ticam2 read through	Negatively regulates MyD88-independent TLR4 pathway
**TOLLIP**	Toll interacting protein	Ubiquitin-binding protein interacts with TLR signalling cascades. Inhibits cell activation by bacterial products. Inhibits IRAK1 phosphorylation.
**TRAF6**	TNF receptor-associated factor 6	Signal transducer in the Toll/IL-1 family and NFκB pathway
**XPO1**	Exportin 1	Mediates export of cellular proteins (mRNA) between the nucleus and cytoplasm. Possible nuclear export inhibitor.
