# Supplementary figures and images for: Differential Effects of MitoVitE, α-Tocopherol and Trolox on Oxidative Stress, Mitochondrial Function and Inflammatory Signalling Pathways in Endothelial Cells Cultured under Conditions Mimicking Sepsis
Source: Antioxidants (Basel). 2020 Feb 26;9(3):195. doi: 10.3390/antiox9030195 (PMC7139367; doi:10.3390/antiox9030195)

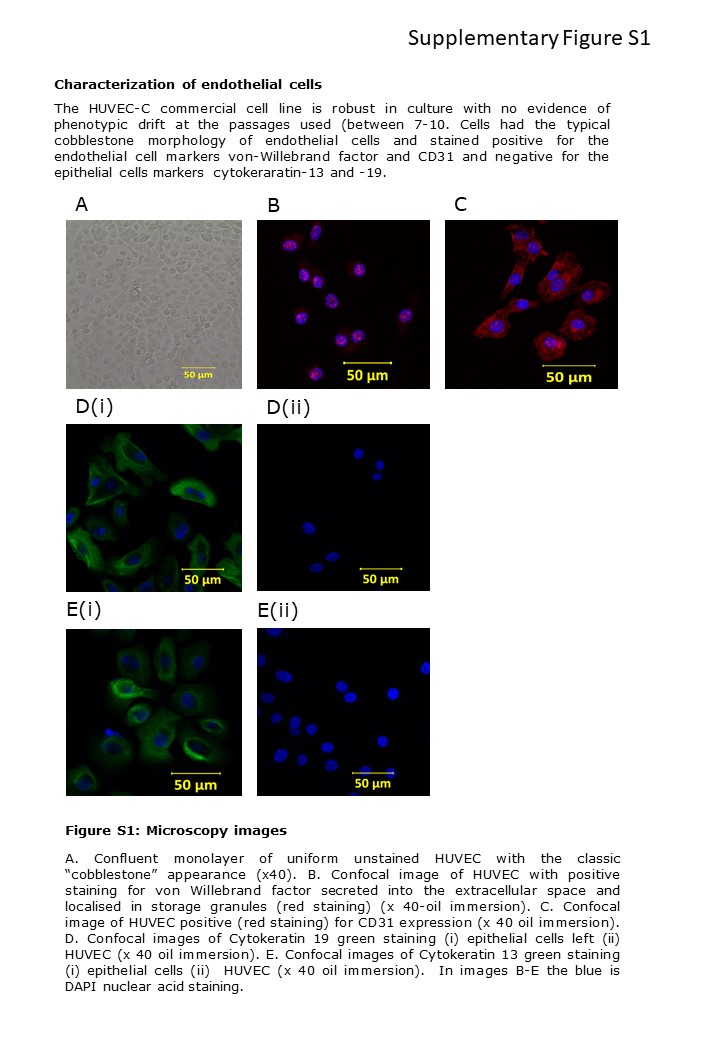

Supplement: Supplementary file 1 [file antioxidants-09-00195-s001.zip › Slide1.JPG]

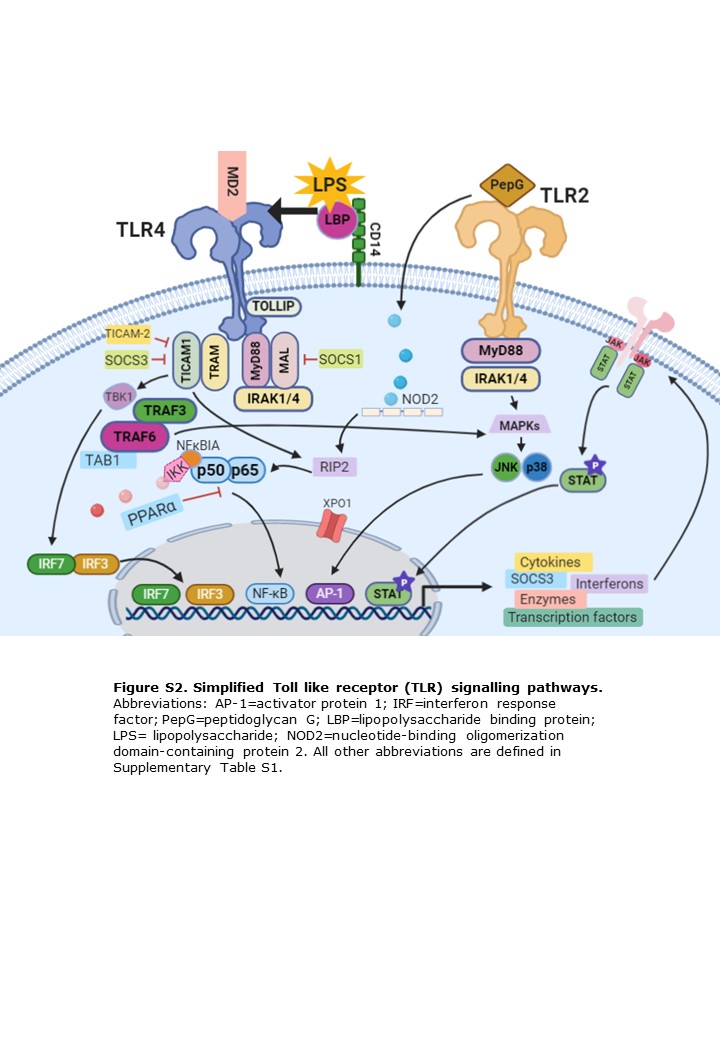

Supplement: Supplementary file 1 [file antioxidants-09-00195-s001.zip › Slide2.JPG]

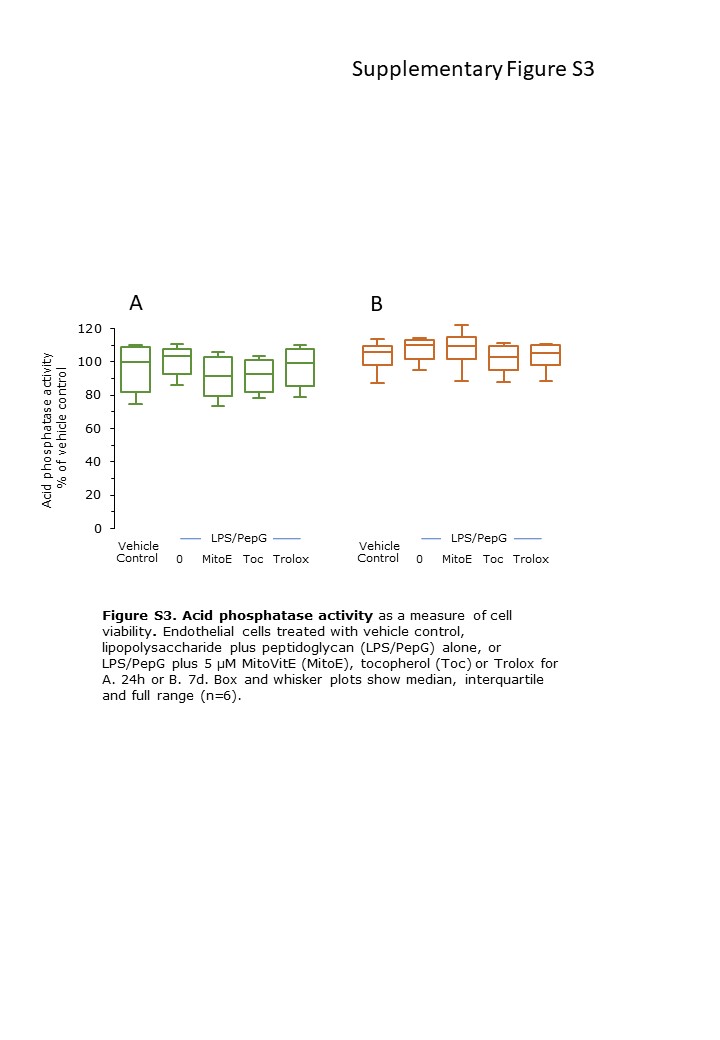

Supplement: Supplementary file 1 [file antioxidants-09-00195-s001.zip › Slide3.JPG]

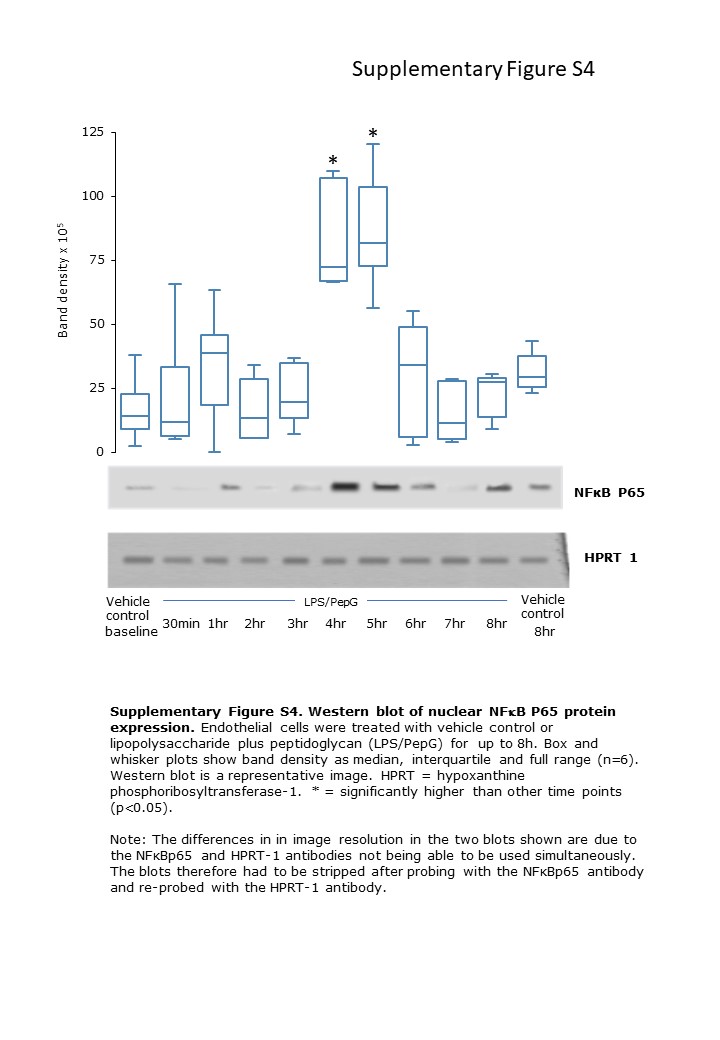

Supplement: Supplementary file 1 [file antioxidants-09-00195-s001.zip › Slide4.JPG]
